# Impact of the huge 2011 Tohoku-oki tsunami on the phenotypes and genotypes of Japanese coastal threespine stickleback populations

**DOI:** 10.1038/s41598-018-20075-z

**Published:** 2018-01-26

**Authors:** Manabu Kume, Seiichi Mori, Jun Kitano, Tetsuya Sumi, Shotaro Nishida

**Affiliations:** 1 Gifu-keizai University, Kitakata 5-50, Ogaki, Gifu, 503-8550 Japan; 2 Division of Ecological Genetics, National Institute of Genetics, Yata 1111, Mishima, Shizuoka, 411-8540 Japan; 3 Daido University, Hakusui 40, Minami, Nagoya, Aichi 457-8532 Japan; 4 Present Address: Field Science Education and Research Center, Kyoto University, Kitashirakawa-Oiwake, Sakyo, Kyoto, Kyoto, 606-8502 Japan

## Abstract

On March 11, 2011, a large earthquake occurred, causing a tsunami which struck the Pacific coast of northeast Japan. We investigated the ecological and genetic effects of the large tsunami on the threespine stickleback (genus *Gasterosteus*) populations in Otsuchi Town, which was one of the most severely damaged areas after the tsunami. Our environmental surveys showed that spring water may have contributed to the habitat recovery. Morphological analysis of the stickleback before and after the tsunami showed morphological shifts in the gill raker number, which is a foraging trait. Genetic analyses revealed that the allelic richness of one population was maintained after the tsunami, whereas that of another decreased in 2012 and then started to recover in 2013. Additionally, we found that the large tsunami and ground subsidence created new spring water-fed pools with sticklebacks, suggesting that the tsunami brought sticklebacks into these pools. Genetic analysis of this population showed that this population might be derived from hybridization between freshwater *Gasterosteus aculeatus* and anadromous *G*. *nipponicus*. Overall, our data indicate that tsunamis can influence morphologies and genetic structures of freshwater fishes. Furthermore, spring water may play important roles in the maintenance and creation of fish habitats, faced with environmental disturbance.

## Introduction

Natural disasters, such as typhoons, volcanic eruptions, earthquakes, and tsunamis, can cause catastrophic damage, not only to human livelihood but also to natural animal and plant populations. Such catastrophic events may lead to drastic changes in the habitat qualities of various organisms, resulting in a decrease or possibly an increase in the numbers of particular species, and also affect dispersal of organisms^1–3^. New habitats can also be created by catastrophic events^4^, where founder organisms can invade and may rapidly change their phenotypes to adapt to the new environments^5–7^. As catastrophic disasters are usually unpredictable, few opportunities are available to investigate their before and after effects of the catastrophic events on natural populations.

Huge tsunamis, triggered by immense earthquakes, are one of the largest catastrophic events and can disturb aquatic and terrestrial ecosystems along coastal areas. In the 21st century, several huge tsunamis have occurred: the 2004 Indian Ocean tsunami, the 2010 Chilean tsunami, and the 2011 Tohoku-oki tsunami. Previous studies have focused on the effects of tsunamis on aquatic ecosystems^8–19^. In these cases, the tsunamis disturbed the habitats of wild aquatic animals by changing landforms, deteriorating water and soil qualities, and bringing in sediments and debris. Tsunami waves also flushed several marine and freshwater organisms and displaced them from their native habitats to non-native places^12,13^. In addition, tsunami waves transported marine materials, such as seawater and sea bottom slime, into freshwater habitats^13–15^, and thereby, freshwater-adapted and stenohaline organisms, including several freshwater fishes, may become extinct in these salinized environments^20^. These effects can lead to biodiversity loss in tsunami-inundated areas.

Although the effects of tsunamis on ecosystems have been investigated, we know little about how they affect the phenotypes and genotypes of organisms. Catastrophic events can reduce the genetic diversity and effective population sizes, thereby, increasing the risk of extinction, as a result of multiple factors, such as inbreeding depression, reduction of the standing genetic variation, and the accumulation of deleterious mutations^21,22^. A tsunami may also induce gene flow between populations. Gene flow can homogenize genetic differentiation between locally-adapted populations and may reduce the mean fitness of each population, although gene flow from different populations can occasionally increase genetic variation and increase the ability of adaptive evolution^23^. Therefore, it is crucial to investigate the changes in genetic structures for a better assessment of the extinction risk of natural populations affected by a tsunami.

**Figure 1:**
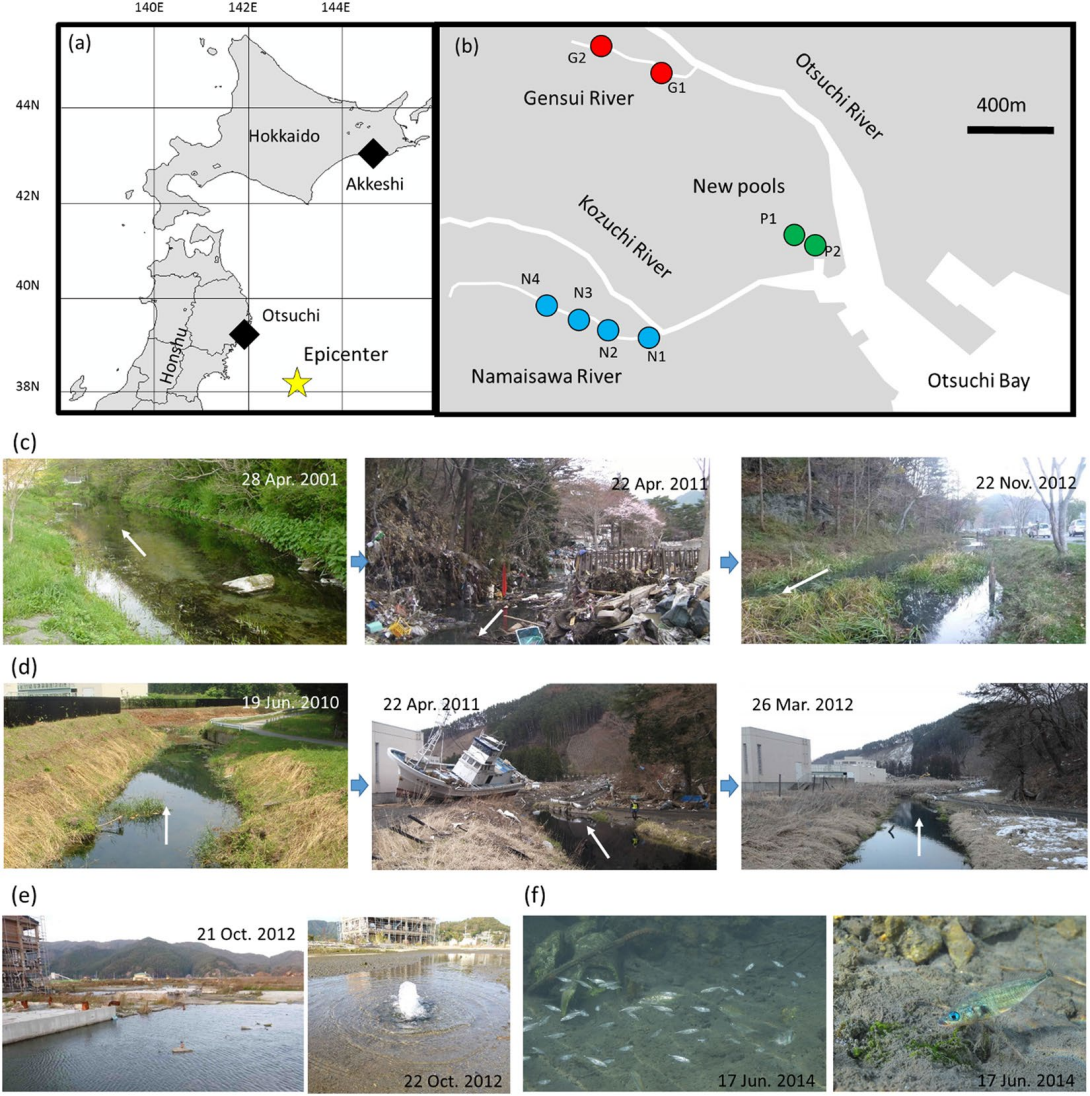
Maps and photographs of the study sites. (**a**) Map of northern Japan. Squares indicate Otsuchi (Iwate Prefecture) and Akkeshi (Hokkaido). The star shows the epicenter of the 2011 off-Pacific coast Tohoku Earthquake. (**b**) Map of Otsuchi Town. Red, blue, and green circles denote survey points of the Gensui River, the Namaisawa River, and the new tsunami-formed pools, respectively. Maps were taken from SAGA (ver. 2.2.7)^60^. Photos of (**c**) the Gensui River and (**d**) the Namaisawa River before and after the 2011 tsunami. Arrows in the photographs show the direction of flow. Photographs (**e**) and (**f**) show a new spring water-fed pool (**e**) and the stickleback inhabiting there (**f**). Photos in Fig. 1f were taken by Mr. Yasuyuki Hata and the other photographs were taken by M.K. and S.M.

On March 11, 2011, the Tohoku Earthquake occurred off of the Pacific coast, registering 9.0 on the Moment Magnitude Scale (epicenter was 38°6′N and 142°51′E; Fig. 1a), and the following tsunami struck the Pacific coast of Honshu and Hokkaido, Japan, especially, in coastal areas of the Tohoku region (Iwate, Miyagi, and Fukushima Prefectures)^24^. Otsuchi Town, which is located at a Pacific coastline of the Iwate Prefecture, Japan, was one of the most severely damaged areas of the 2011 tsunami (Fig. 1 and Supplementary Fig. S1). The urban area of this town is located at an alluvial plain between the Otsuchi River (approximately 27.6 km long) and the Kozuchi River (approximately 26.4 km long). Since a river levee of the Otsuchi River was broken by the tsunami, overflowing water flooded the urban area (Supplementary Fig. S1b).

The present study aims to elucidate the ecological and genetic effects of a large tsunami on the threespine stickleback (genus *Gasterosteus*) in Otsuchi Town. The threespine stickleback is a small cold-water fish, which is widely distributed in the coastal regions of the Northern Hemisphere^25^. The threespine stickleback has both marine/anadromous and freshwater resident-life histories; the ancestral marine/anadromous sticklebacks have colonized freshwater repeatedly in widespread regions, resulting in phenotypic diversification and the evolution of diverse ecotypes^26,27^. In addition, this fish has many types of species pairs^27–29^. Thus, the threespine stickleback is considered a model for evolution, ecological, and genetic research. In Japan, there is a unique species pair composed of *G*. *aculeatus* and *G*. *nipponicus* (corresponding to the Pacific Ocean and Japan Sea sticklebacks in several previous reports, respectively)^30^. These two species are thought to have diverged about 1.0 million years ago^31,32^. These sticklebacks are sympatric in several habitats and reproductively isolated with low levels of on-going hybridization. They have different life history characteristics; some *G*. *aculeatus* are freshwater residents and others are anadromous, while *G*. *nipponicus* is exclusively marine or anadromous^31–34^.

A coastal area of Otsuchi Town is the southernmost sympatric area of the Japanese threespine stickleback pair reported thus far^35^. In this town, there are two main habitats of the freshwater threespine stickleback, which are the spring water-fed tributaries of the Otsuchi and Kozuchi Rivers (Fig. 1). Only freshwater populations of *G*. *aculeatus* live in the tributary of the Otsuchi River, while both freshwater *G*. *aculeatus* and anadromous *G*. *nipponicus* populations co-occur in the tributary of the Kozuchi River. Freshwater stickleback populations were listed as endangered species in the Iwate Prefecture^36^. One population in a tributary of the Otsuchi River, the Gensui population, has been named a natural monument of Otsuchi Town since 2007^37^, so the local citizens have conserved the stickleback as a symbol of biodiversity^35^. When the 2011 tsunami occurred, all of the stickleback habitats in this area were damaged. The tsunami waves broke the river levee of the lower reach of the Otsuchi River (arrow in Supplementary Fig. S1b) and flowed into landside urban areas. All of the stickleback habitats in this area were also severely damaged. For example, huge amounts of debris accumulated on these watersheds, and oil spilled into the main rivers and their tributaries (middle panels in Fig. 1c and d). Within three months, however, the majority of debris and oil were removed by humans^35,38^. Furthermore, spring water supplied clean water and removed residual oil and debris (right panels in Fig. 1c and d); as a result, surviving sticklebacks were found. Another habitat change produced by the tsunami was the appearance of new spring water-fed pools in a coastal area that was previously a downtown area (Fig. 1b and e); these pools were formed by the big tsunami and ground subsidence. In 2012, we found sticklebacks in these newly-formed pools (Fig. 1f), suggesting that the tsunami brought sticklebacks into these pools.

Here, we first surveyed the recovery of the water quality of stickleback habitats after the 2011 tsunami. Second, we compared stickleback morphological traits before and after the tsunami, taking advantage of the fact that we sampled these populations in the year before the tsunami. Third, we investigated changes in the genetic diversity of these populations before and after the tsunami. Finally, we analyzed the genetic structure of the population in the newly formed pools.

## Results

### Recovery of stickleback habitats after the tsunami

**Figure 2:**
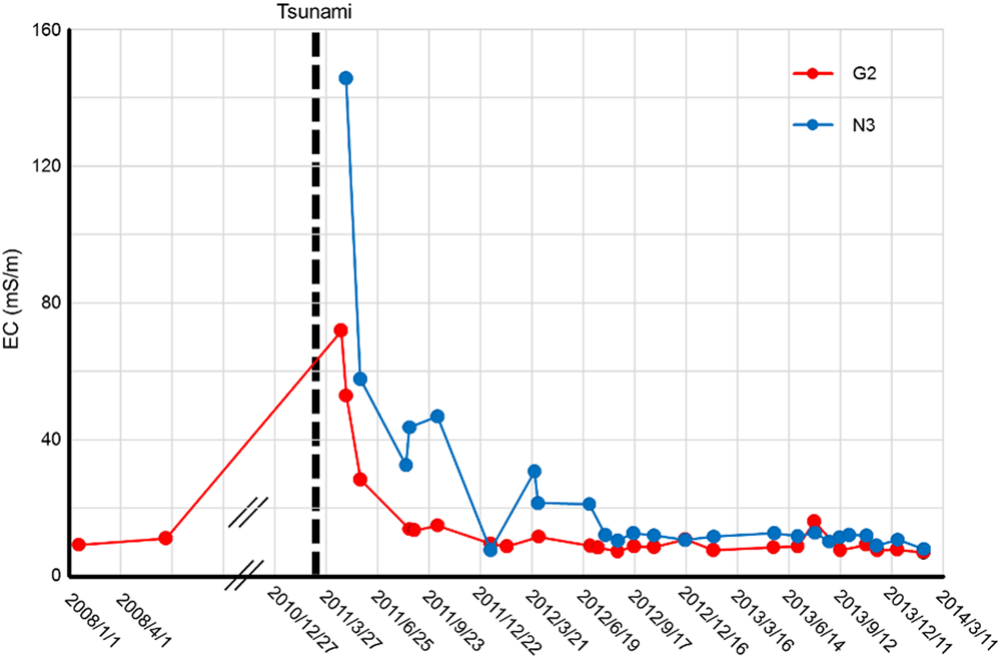
Changes in the electrical conductivity (mS/m) of the Gensui and Namaisawa Rivers. The broken line indicates the timing of the 2011 off-Pacific coast Tohoku Earthquake (March 11, 2011).

In the Gensui River (a tributary of the Otsuchi River; Fig. 1), natural springs from unconfined groundwater degraded in quantity and quality after the 2011 tsunami. Immediately after removal of the sediment of seabed materials and debris brought in by the tsunami (Fig. 1c), the flux quantity recovered from 0.016 m^3^/s on May 1, 2011, to 0.069 m^3^/s on August 16, 2012, at Station G2 (see Fig. 1b). We measured the electrical conductivity (EC) to investigate the invasion of seawater and the recovery of the freshwater. EC is generally positively correlated with ion concentrations (i.e., NO_3_^−^, Na^+^, and Cl^−^) and typically ranges from 9.0 to 11.0 mS/m in this river (Fig. 2 and Supplementary Table S1). EC values increased soon after the tsunami. After 2.5 years, the EC values gradually decreased and recovered to the level prior to the tsunami (Fig. 2), suggesting that underground spring waters steadily flushed the seawater brought in by the tsunami. After late-May 2011, the sediment of seabed material and debris was removed by volunteers^35,38^, and thereby, we could find adult sticklebacks in the Gensui River during this time period (T. Sumi, pers. obs.); at the present time, large numbers of mature adult sticklebacks and their offspring are found every year.

Patterns of the temporal change in the EC values of the Namaisawa River (a tributary of the Kozuchi River; Fig. 1) showed a similar tendency with that of the Gensui River (Fig. 2). In this river, spilled oil was one of the critical causes of the deterioration of water and riverbed quality (Fig. 1d). Soon after the 2011 tsunami, an oil fence was constructed above a survey point of N3, which prevented the oil from floating to the surface and downstream; this was followed by oil removal by humans. At the same time, deposited debris was removed by humans, resulting in the gradual recovery of the EC values, where the EC values may represent both the oil quantity and salinity level. The water quality of the Namaisawa River also recovered to the level prior to the tsunami 2.5 years later. However, it should be noted that oil still remains in the riverbed of the lower reach (N1) even today (M. Kume & S. Nishida, pers. obs.). At the middle reaches of this river (N3 and N4), we could find sticklebacks, some of which were nesting after July 2011 (S. Mori & M. Kume, pers. obs.).

### Morphological changes

**Figure 3:**
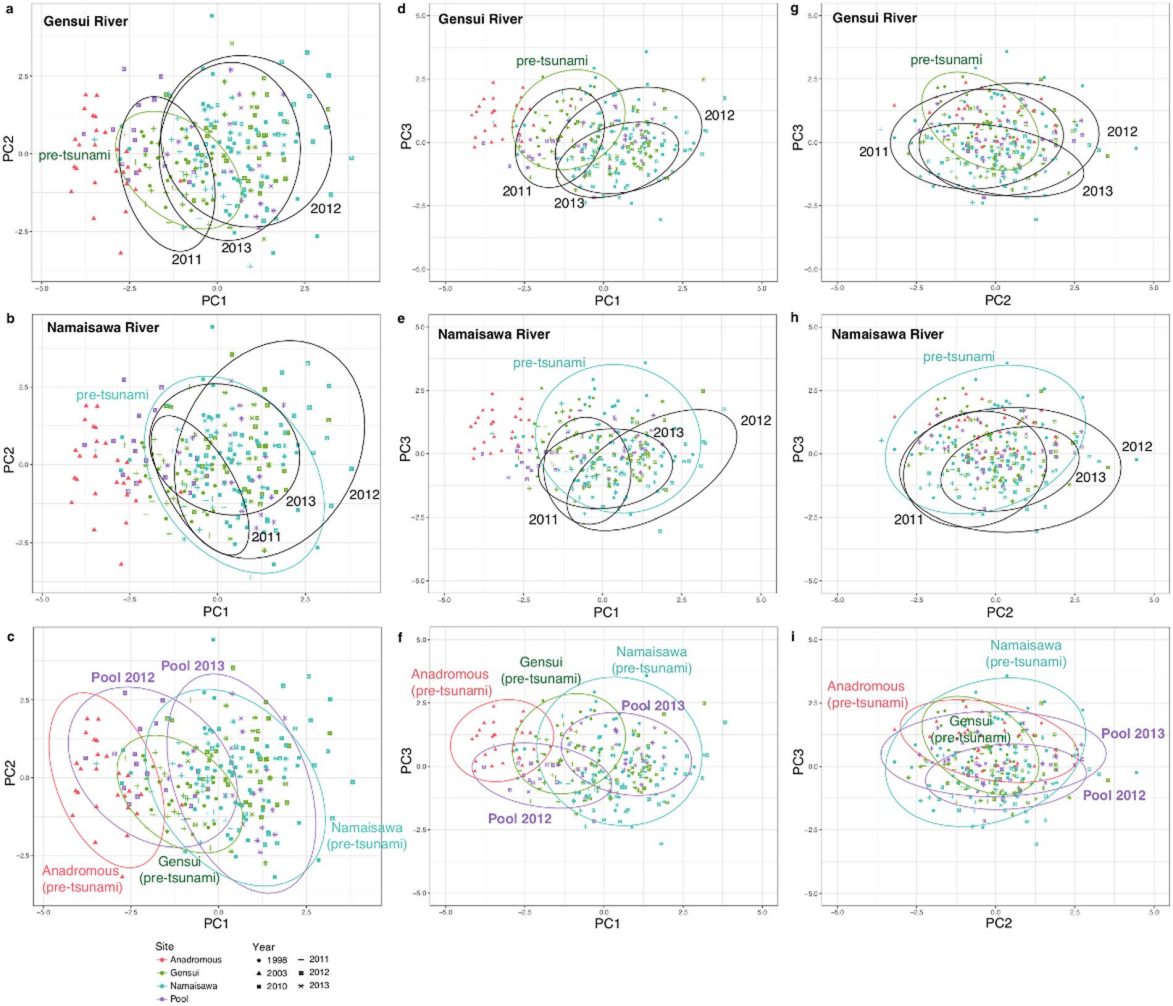
Results of the PC analyses. Scatter plots of (**a–c**) PC1 and PC2, (**d–f**) PC1 and PC3, and (**g–i**) PC2 and PC3 of the body size-corrected morphological traits. The circles are 95% confidence ellipses. The upper panels (**a,d,** and **g**) indicate morphological changes in the Gensui population. The middle panels (**b,e,** and **h**) show morphological changes in the Namaisawa population. The lower panels (**c,e,** and **i**) compare the morphologies of the new pool population (Pool 2012 and Pool 2013) with those of the anadromous and the native Gensui and Namaisawa populations before the tsunami.

Principal component (PC) analysis of the morphological traits of the adult sticklebacks was conducted, following body size correction of the external morphological traits, using the standard length (Table 1 and Fig. 3). The first three PCs explained 68.8% of the total variance (Table 1): a higher PC1 (27.75%) reflects a deeper body and a shorter dorsal spine, a higher PC2 (24.45%) indicates a smaller head and a shorter pelvic spine, and a higher PC3 (16.57%) indicates a slender and larger eye.

**Table 1:** Principal component (PC) analysis of morphological traits.

Component loadings	PC1	PC2	PC3
Head length	0.2588	−0.5672	0.2516
Body depth	0.4694	−0.0372	−0.4509
Caudal depth	0.3991	−0.3201	−0.2781
Eye diameter	0.2818	−0.3922	0.4906
2nd dorsal spine length	−0.4822	−0.3655	−0.3434
Left pelvic spine length	−0.3335	−0.5136	−0.3307
Gill raker number	−0.3610	−0.1522	0.4337
Eigenvalues	1.94	1.71	1.16
% Variance explained	27.75	24.45	16.57

Scatter plots with 95% confidence ellipses showed that both the Gensui and Namaisawa populations changed morphology in all three of the PC axes across years (Fig. 3). The year effect was significant for each population (ANOVA, *P* < 0.0001). Interestingly, all PC scores showed similar temporal changes in both the Gensui and Namaisawa populations (comparison between Fig. 3a and b, Fig. 3d and e, and Fig. 3g and h). A general linear model, including both populations, showed that interactions between the year and population were not significant for PC2 (*P* = 0.6398) and PC3 (*P* = 0.1736), whereas the effect of the year was significant in both PCs (Supplementary Table S3), suggesting that the yearly change patterns were similar between populations. Although PC1 showed a significant interaction between the year and population (*P* = 0.00256; Supplementary Table S3), both populations showed a relatively similar trend for the change in PC1; PC1 increased in 2012 and decreased in 2013. After the tsunami, PC1 and PC2 increased in both populations, indicating that the post-tsunami fish have deeper bodies, shorter dorsal and pelvic spines, and smaller heads than the pre-tusnami fish. PC3 decreased, indicating that the post-tsunami fish have smaller eyes and deeper bodies than the pre-tsunami fish.

Next, we analyzed the morphology of the newly formed pool population (Fig. 3c,f, and i). PC1 and PC3 differed between the sampling years (ANOVA, *P* < 0.0001 for PC1 and *P* = 0.0001 for PC3), while PC2 did not differ between 2012 and 2013 (*P* = 0.265). A scatterplot of PC1 and PC3 (Fig. 3 f) showed that the new pool population was similar to the anadromous population in 2012, but shifted toward the Namaisawa population in 2013. Generally, the morphology of the new population was included within the total variations of the anadromous and freshwater populations (Fig. 3c,f, and i). This new pool population may be derived from hybridization between the anadromous *G*. *nipponicus* and freshwater *G*. *aculeatus* populations (see below). Since hybridization can increase the phenotypic variance and change the phenotypic variance-covariance matrix^39,40^, we calculated the ratio between the length of the major and minor axis of the ellipse (eccentricity) and the size of the ellipse (Supplementary Fig. S3). We did not observe any clear increase in the size or decrease in the eccentricity in the new pool population, compared to the native populations; the values were within the range of the other native populations (Supplementary Fig. S3).

**Figure 4:**
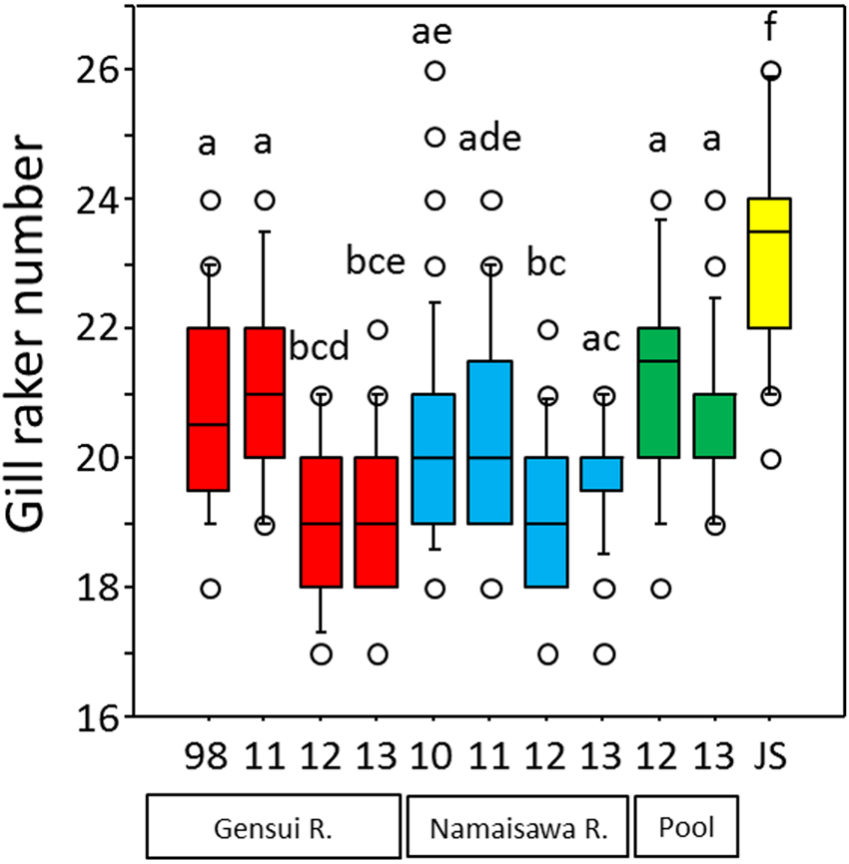
Box plots of the gill raker number. Letters above the boxes indicate the results of the pairwise *post-hoc* Mann-Whitney *U*-tests with Bonferroni corrections (*P* < 0.0009): the same letters indicate the absence of significance in the pairwise comparison, whereas different letters indicate significant differences between groups with different letters. Specimens in 1998 and 2010 were collected before the tsunami, whereas those in 2011–2013 were collected after the tsunami. JS denotes anadromous populations of *G*. *nipponicus* caught in 2003.

The gill raker number is one of the important foraging traits^25–29^, and our previous studies showed significant variations in the gill raker number among Japanese populations^32^, so we further analyzed this trait (Fig. 4). The gill raker numbers were significantly different among populations across years (Kruskal-Wallis test, χ^2^ = 98.45, *P* < 0.0001); both the Gensui and Namaisawa populations in 2012 and 2013 had smaller gill raker numbers, than those populations before 2011 and the 2012 and 2013 new-pool populations (*post hoc* test, *P* = 0.0027–0.1511 for the 2013 Namaisawa population vs. the other Namaisawa and pool populations, *P* = 0.0009 for the 2011 vs. 2012 Namaisawa populations, *P* < 0.0009 for others). However, there was no significant difference between 2012 and 2013 in the new-pool populations (*P* = 0.2807; Fig. 4 and Supplementary Table S2).

### Changes in the allelic richness and analysis of genetic structures

**Figure 5:**
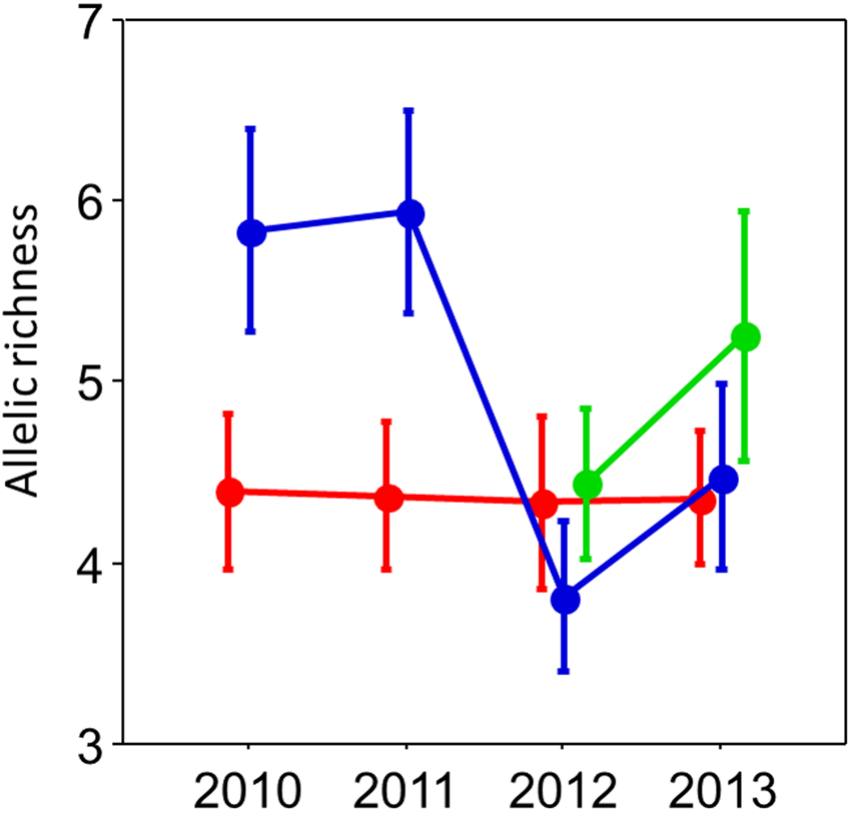
Allelic richness (mean ± SE) across the years, measured with 15 microsatellite loci. Red, blue, and green lines indicate the Gensui, Namaisawa and new-pool populations, respectively. Specimens in 2010 were collected before the tsunami, whereas those in 2011–2013 were collected after the tsunami. The allelic richness of each locus is shown in Supplementary Table S4.

Overall, the Gensui population has a low allelic richness, but no substantial reduction of the allelic richness occurred after the tsunami (Friedman test, χ^2^ = 0.680, *P* = 0.8779; Fig. 5 and Supplementary Table S4). The allelic richness of the Namaisawa population (χ^2^ = 30.67, *P* < 0.0001) showed a substantial reduction in 2012 (*post hoc* test, *P* = 0.0007) and recovered slightly in 2013 (*P* = 0.0231; Fig. 5 and Supplementary Table S4). The allelic richness of the new pool population showed a similar trend of an increase in the allelic richness in 2013 (Wilcoxon signed ranks test, *z* = −1.915, *P* = 0.055; Fig. 5 and Supplementary Table S4).

**Figure 6:**
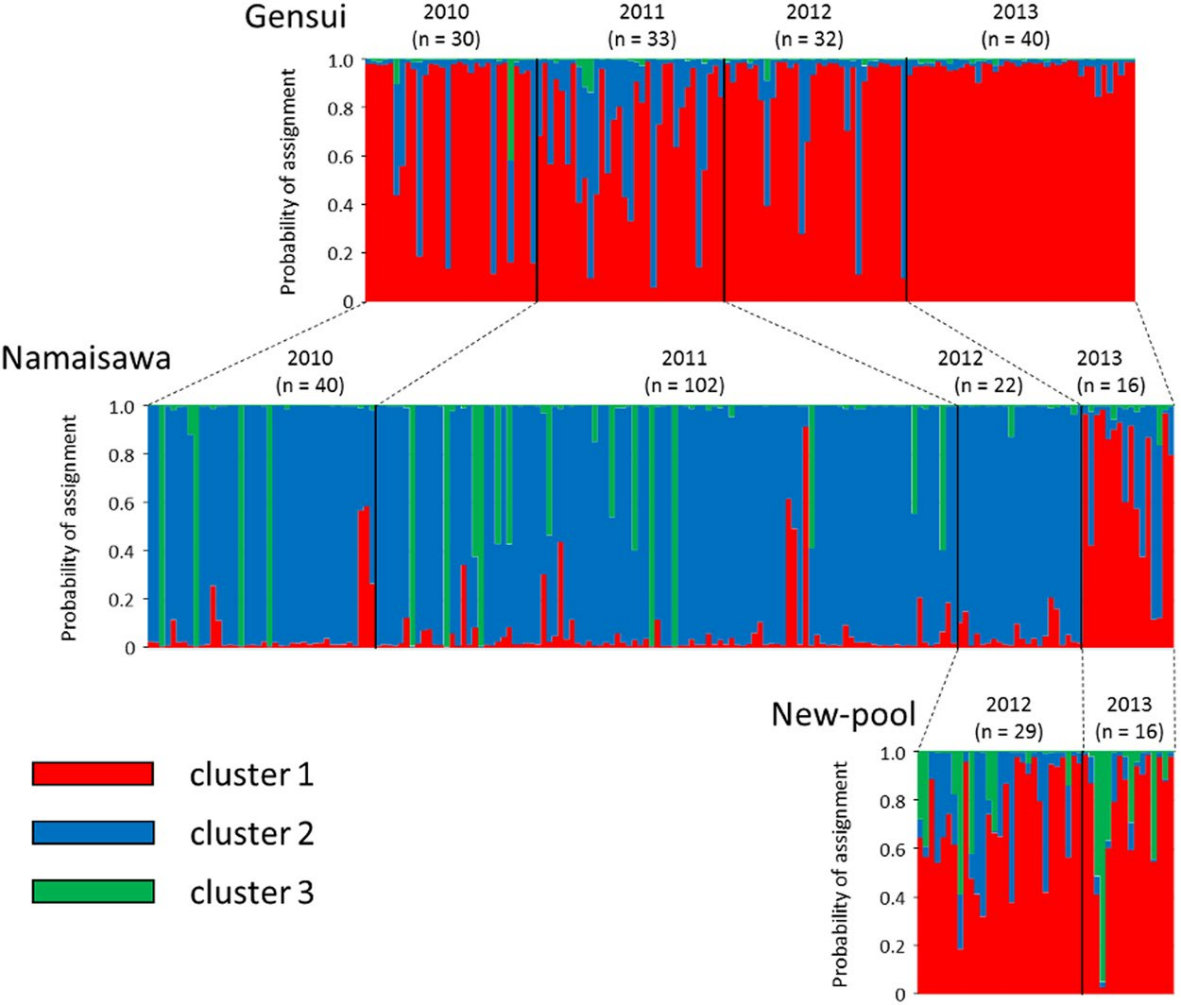
Genetic structure of threespine sticklebacks collected from Otsuchi during 2010–2013. The probability of assignment to each genetic cluster estimated by STRUCTURE with *K* = 3 (see also Supplementary Fig. S4). *n* indicates the sample size. Specimens in 2010 were collected before the tsunami, whereas those in 2011–2013 were collected after the tsunami.

STRUCTURE analysis revealed that there are three genetically distinct clusters among these populations from Otsuchi Town; both Evanno’s Δ*K* and L(*K*) indicated that the most likely cluster number was three (*K* = 3) (Supplementary Fig. S4). The Gensui population is predominantly composed of cluster 1, whereas the Namaisawa population is mainly composed of cluster 2 (Fig. 6). Cluster 3 likely represents *G*. *nipponicus*, because genotypes for the species-diagnostic microsatellite markers showed that the fish belonging to cluster 3 possessed *G*. *nipponicus*-specific lengths of microsatellite markers^33^. In 2010 and 2011, we found several fishes (four in 2010 and five in 2011) of *G*. *nipponicus* in the Namaisawa River (nearly 100% cluster 3 assignment), but no *G*. *nipponicus* was observed in 2012 and 2013 (Fig. 6), which may be the reason why the allelic richness decreased in 2012 and 2013. The newly formed pool population is composed of cluster 1, cluster 3, and their mixture (Fig. 6). In 2013, one fish of the new pool population belonged to cluster 3, namely *G*. *nipponicus*.

## Discussion

Immediately after the 2011 tsunami, the water quality of the main rivers and their tributaries in Otsuchi Town were deteriorated because of several factors, including sea water and marine slime brought in from the sea and chemicals released from debris and deposits. Such cases occurred along the Japanese coastline across the 2011 tsunami-struck areas^13,14,41^. In the Gensui River, however, debris and slime were removed by humans^35,38^, which may have helped recover the discharge of spring water and the survival of the stickleback. In another stickleback habitat, the Namaisawa River, river water was polluted by inflowing oil, but the oil was removed by both humans and clean water supplied by the natural flow of river and spring waters, which may also have helped the survival of *G*. *aculeatus*. We confirmed that the stickleback was breeding in the mid-upper reaches (N3 and N4) in July of 2011 (S. Mori & M. Kume, pers. obs.). In both places, therefore, immediate removal of debris by humans and a continuous supply of spring water potentially played a role in the recovery of the stickleback habitats. This idea is supported by our data of the temporal change in EC (Fig. 2 and Supplementary Table S1). However, further study is needed to understand the tsunami’s long-term effects on the riverine ecosystem, because large earthquakes and tsunamis may continuously influence the quality of the groundwater, not only for a short-term but also for medium- and/or longer-terms^42,43^.

We found morphological shifts in both the Gensui and Namaisawa populations after the tsunami (Table 1 and Fig. 3a). One of the remarkable changes was found in a feeding trait, the gill raker number, which significantly reduced in both populations after 2012 (Fig. 4 and Supplementary Table S2). Fish collected in 2012 are likely the generations that were born after the 2011 tsunami. In general, fish with a greater gill raker number are planktivorous, whereas fish with a lower gill raker number are benthivorous^27^. The observed difference in the gill raker number was 2–3, and this amount of difference can be found between stickleback ecotypes^44,45^. Therefore, it is possible that these morphological changes occurred as an adaptation to the tsunami-induced changes in the available food items. However, sympatric ecotypes usually differ by 3–6 gill raker numbers, probably because of character displacement^46^. In addition, we could not directly compare the food items between before and after the tsunami, due to the lack of stickleback’s prey item data before the tsunami. Therefore, further detailed, long-term analysis of temporal changes in the available food items, which we initiated just after the tsunami, should be continued to understand the ecological correlates of this phenotypic shift.

Our genetic analysis revealed that the genetic diversity of the Gensui population did not reduce after the tsunami (Fig. 5). There may be two reasons why this freshwater population could maintain its genetic diversity after the tsunami. One is that the quality and quantity of the river and spring waters recovered fairly quickly after the tsunami disaster, as described above. Another reason may be that the Gensui population of *G*. *aculeatus* may have a relatively high salinity tolerance. Although a previous experimental study showed that all individuals of a freshwater population of a Canadian river died within 12 h of exposure to sea water^47^, our preliminary seawater challenge experiment, using the Gensui population, indicated that there was no significant difference in the survival rate over 24 h between fish exposed to seawater (mean 33.75 psu) and to freshwater (M. Kume, unpubl. data).

In contrast, the allelic richness of the Namaisawa population reduced in 2012 (Fig. 5). This reduction might be due to the reduction in spawning migration of *G*. *nipponicus* (cluster 3). In fact, our genetic results revealed that *G*. *nipponicus* migrated into the Namaisawa River before the 2011 tsunami, but not after the tsunami (Fig. 6). A reduction in the spawning migration of *G*. *nipponicus* may be due to the deterioration of the spawning habitats. Alternatively, the number of migratory sticklebacks may be simply fluctuating from year-to-year, as observed in the Bekanbeushi River in Hokkaido^48^. As we released the majority of the caught fish, we believe that our sampling in 2011 did not affect the allelic decline observed in the next year (2012). In 2013, fish belonging to cluster 1 increased, although the reason for this increase is currently unclear. Further long-term monitoring of the number of anadromous migrants, the allelic richness, and the genetic structures will help to identify the factors that affect the genetic structure in this population.

We found that the tsunami created new stickleback habitats (Fig. 1e). New pools were created by underground spring water that appeared in a coastal urban area (Supplementary Fig. S2); furthermore, this area showed ground subsidence of 300–600 mm from the 2011 earthquake, which allowed the inundation of seawater (T. Sumi, unpubl. data). Our genetic analysis suggested that the new-pool population mainly consisted of a cluster similar to the Gensui population (cluster 1), a cluster corresponding to *G*. *nipponicus* (cluster 3), and their hybrids (Fig. 6). Our hypothesis for the formation of new threespine stickleback populations in the tsunami-formed pools is as follows. First, new pools were formed by the ground subsidence and inundation of seawater. Some freshwater *G*. *aculeatus* were brought in by the backwash of the tsunami waves from the Gensui River. *G*. *nipponicus* may have been brought in from the sea by the tsunami or they migrated to these pools. The P2 pool is always connected to the Kozuchi River through a flood gate. The P1 is usually isolated from the sea, but it is sometimes connected with P2 through a narrow channel (approximately 1 m width), when the water levels of the rivers and pools rise due to spray tides and rainfall, which enables anadromous sticklebacks to migrate into these pools. These new pools have been maintained through present day by discharging spring water that comes from flowing wells.

Our genetic data indicate that the freshwater population of *G*. *aculeatus* and anadromous *G*. *nipponicus* seem to be hybridizing in the new tsunami-formed pools (hybrids between cluster 1 and cluster 3). Hybridization can often increase phenotypic diversity and change the phenotypic variance-covariance matrix, which may help the hybrid populations adapt to new environments^39,40^. Our analysis of the new pool population did not show any apparent changes in the variances and the phenotypic matrix. However, further analysis of the morphological changes would be necessary to make any conclusions about the roles of hybridization in adaptation to the new pool habitats. Recently, stickleback adaptations to newly formed environments, following large earthquakes and tsunamis in the wild, were investigated in a different region, but the formation processes of the new habitats and populations were different. A previous study showed that new habitats were formed because of an island uplift, which was caused by the 1964 Great Alaskan Earthquake, and then anadromous *G*. *aculeatus* stickleback invaded the new habitats^5,7^. In our case, new habitats were formed by ground subsidence and spring water, and both anadromous *G*. *nipponicus* and freshwater *G*. *aculeatus* sticklebacks invaded. Thus, the new pool population in Otsuchi provides an opportunity to investigate mechanisms by which freshwater and coastal fishes disperse, hybridize, and adapt to new environments.

There is a possibility that the immediate removal of debris and oil by humans might help maintain freshwater fish habitats. In addition, spring waters might also play important roles in the maintenance of animal populations, faced with environmental disturbances from natural disasters. If so, conservation of spring water would be key for recovery of the aquatic ecosystem. Finally, it should be noted that local citizens used groundwater for human livelihood, when they could not use the public water system, suggesting that ground/spring waters are important for human beings, as an alternative water resource during emergencies^38,49^. However, it is worth noting that recovery projects, including natural disaster contingency planning, are now being performed in Otsuchi Town^50^. These activities may ironically threaten various ecosystems. For example, construction of temporary houses, after landfills of spring water (Supplementary Fig. S2), may lead to habitat destruction. Thus, discussion between biologists and policy makers should be continued during long-term town recovery planning, because it will be essential for conserving aquatic biodiversity and sustaining the ecosystem functions of spring/ground waters.

## Methods

### Ethics statement

All fieldwork was performed in accordance with local ethical regulations and agreements. All procedures were approved by the institutional animal care and use committee of the National Institute of Genetics (23-15, 24-15, 25-18). All experiments were performed in accordance with relevant guidelines and regulations.

### Study site

We conducted field surveys at the Gensui River (approximately 450 m long, mean 3.73 m width, mean 33 cm depth), which is a tributary of the Otsuchi River, and the Namaisawa River (approximately 2280 m long, mean 4.71 m width, mean 76 cm depth), which is a tributary of the Kozuchi River; both rivers run through a lowland area of Otsuchi Town (39°21′N, 141°54′E), Iwate Prefecture, Japan (Fig. 1). We set two and four survey points in the Gensui River (G1 and G2) and the Namaisawa River (N1 to N4), respectively (Fig. 1b). Downstream of the Namaisawa River (N1 to N2) was tidal areas, while the other survey points were always freshwater (Supplementary Table S1). According to our previous studies^35^, only the freshwater-resident *G*. *aculeatus* inhabits the Gensui River, while both freshwater-resident *G*. *aculeatus* and anadromous *G*. *nipponicus* were found in the Namaisawa River.

The urban area of Otsuchi Town is a lowland area with abundant groundwater, which people have used for their daily life (Supplementary Fig. S2)^38,51^. During our study, on July 22, 2012, we found threespine sticklebacks in new spring water pools in the urban area for the first time, so we have no samples before that time. We have sampled sticklebacks in these two pools (P1 and P2; Fig. 1b) since then.

### Environmental measurement

We measured five parameters of water quality at all of the sites of the Gensui and Namaisawa Rivers after the 2011 tsunami; water temperature (WT, °C), electrical conductivity (EC, mS/m) and pH were recorded using a multi-parameter water quality probe (WM-22EP, DKK-TOA Co. Ltd., Tokyo, Japan). These measurements were conducted nearly every month from April 2011 to August 2014. Before the 2011 earthquake, we had measured some of these parameters in both rivers. Also, we started these environmental measurements in the tsunami-formed pools (P1 and P2) after July 2012.

### Fish sampling

Samples were collected using minnow traps for adults and dip-nets for juveniles. Adult fish used for morphological analyses were sampled from the Gensui River at survey site G2 on December 22, 1998, December 24, 2011, July 1, 2012, and May 19, 2013; from the Namaisawa River at survey site N3 on June 26, 2010, December 24, 2011, July 29, 2012, and May 20, 2013; and from the new-pool at survey site P1 on July 29, 2012 and May 19, 2013 (Table 2). Fish were preserved in ethanol after euthanasia. Fish collected from G2 on December 24, 2011, and from N3 on December 24, 2011, were adults born after the tsunami.

**Table 2:** Summary of sample sizes.

Population/year	Sample size		
	Morphological analyses	Genetic analyses	All specimens
Gensui			
1998	20	0	20
2010	0	30	30
2011	20	33	53
2012	28	32	60
2013	20	40	60
Namaisawa			
2010	41	40	41
2011	20	102*	122
2012	26	22	48
2013	20	16	36
New-pool			
2012	18	29	47
2013	20	16	36
Anadromous			
2003	26	—	26

*55 and 47 fish were adults and juveniles, respectively.

Adult threespine stickleback used for genetic analysis were collected from the Gensui (G2) and Namaisawa Rivers (N3) on June 26, 2010, May 26, 2011, August 21, 2012, and June 29–30, 2013, (Table 2) using minnow traps. Adults collected from G2 and N3 on May 26, 2011 were born in 2010. Juvenile fish (sample size = 47) were also sampled from the Namaisawa River (N3) on July 9, 2011 (Table 2). Fish of the new pool (P1) were sampled on August 21, 2012, and Feb 2, 2013 (Table 2). After euthanasia, pectoral or caudal fins were cut and saved in ethanol, except for the 2010 Gensui population, for which small tips of fins were cut and stored in ethanol after anesthesia, and the fish were released to the river after they recovered from the anesthesia.

### Morphological measurements

We used 233 adult sticklebacks (range: 18–41 for each group) for the morphological analyses (Table 2), and their standard lengths ranged from 45.49–67.12 mm in 1998, 40.14–64.48 mm in 2011, 38.42–70.78 mm in 2012, and 49.35–66.75 mm in 2013 for the Gensui population; 41.92–79.69 mm in 2010, 42.95–51.62 mm in 2011, 47.57–60.57 mm in 2012, and 46.52–68.30 mm in 2013 for the Namaisawa population; and 34.57–44.10 mm in 2012 and 46.90–59.49 mm in 2013 for the new-pool population. An anadromous population of *G*. *nipponicus* was collected from the Bekanbeushi River, Akkeshi, Hokkaido, Japan in 2003^32,48^ and preserved in 10% formalin until the morphological analysis. We measured the standard length, head length, body depth, caudal depth, second dorsal spine length, left and right pelvic spine length using a digital caliper (nearest 0.01 mm), and counted the gill raker number using a dissecting microscope (Supplementary Table S2). In this study, plate morph was not analyzed, because all sticklebacks analyzed here were completely plated. We analyzed morphological variations, using a principal component (PC) analysis with a correlation matrix. Standard length is commonly correlated with other size traits. We therefore used the residuals of the linear regressions of the ln-transformed morphological trait values, except the gill raker number, against the ln-transformed standard length. For regression, all fish were pooled. The gill raker number is independent of the body size^32^, so it was just ln-transformed before PC analysis. We first performed PC analysis excluding the anadromous population. Next, the PC scores of the anadromous population were calculated using the *predict* function in *prcomp*. We used a general linear model for the statistical analysis of the PC scores. The 95% confidence ellipse was drawn with the *stat_ellipse* function in *ggplot*. The eccentricity and size of the phenotypic matrix were calculated using the R package “car”. In order to investigate divergence in a foraging trait, the gill raker number, we conducted the Kruskal-Wallis test (α = 0.05), followed by pairwise *post-hoc* Mann-Whitney *U*-tests with Bonferroni corrections (α = 0.0009). These analyses were performed in R version 3.0.2^52^.

### Genetic analyses

We used 360 sticklebacks (range: 16–102 for each habitat) for the genetic analyses (Table 2). Genomic DNA was isolated with the Qiagen DNeasy Blood & Tissue Kit (Qiagen, Valencia, CA, USA). For genetic analysis, fourteen microsatellite markers, which are located on different linkage groups and are not linked to sex, were used, as described previously (Supplementary Table S4)^53^: *Stn90*, *Stn64*, *Stn159*, *Stn46*, *Stn120*, *Stn384*, *Stn332*, *Stn278*, *Stn76*, *Stn170*, *Stn175*, *Stn301*, *Stn389*, *Stn25*, and *Stn35*. Forward primers were labeled with fluorescence (HEX, NED, or FAM), and the 5′-end of the reverse primers were tailed with GTTTCTT to increase the accuracy of the fragment length analysis^54^. Microsatellites were amplified with three combinations of primer sets, with three different dye colors, using the KAPA2G Fast Multiplex PCR Kit (KAPA Biosystems, Woburn, MA, USA). After 3 min at 95 °C, 30 cycles of 95 °C for 15 sec, 60 °C for 30 s, and 72 °C for 30 s were performed, followed by 10 min at 72 °C. Amplified fragments were analyzed by BEX Co. Ltd. (Tokyo, Japan). Allele lengths were then determined using Peak Scanner Software (Life Technologies, Grand Island, NY, USA). Micro-Checker was used to confirm the accuracy of genotyping^55^.

Data were first analyzed using STRUCTURE, which uses Markov chain Monte Carlo simulations to identify groupings that minimize Hardy-Weinberg and linkage disequilibrium within cluster groups^56^. Five simulations were run for each cluster number (*K*) from *K* = 1 through *K* = 10. We estimated the probable number of clusters by finding the *K* value with the highest log likelihood Ln(*K*) and the *K* value with the highest Δ*K*, which is the rate of change of Ln(*K*) between successive *K* values^57^. This analysis was performed using Structure Harvester^58^. Parameters were estimated after 500,000 iterations, following a burn-in of 50,000 iterations. The allelic richness (number of alleles per locus, corrected for sample size) was calculated using FSTAT 2.9.3 software^59^. In order to confirm yearly changes in the allelic richness within each population, we conducted the Friedman test (α = 0.05), followed by pairwise *post hoc* Wilcoxon signed ranks tests with Bonferroni corrections (α = 0.0083), and Wilcoxon signed ranks test (α = 0.05). These analyses were performed in R version 3.0.2.^52^.

## Electronic supplementary material

Supplementary Information
