# Supplementary material for: Impact of the huge 2011 Tohoku-oki tsunami on the phenotypes and genotypes of Japanese coastal threespine stickleback populations
Source: Sci Rep. 2018 Jan 26;8:1684. doi: 10.1038/s41598-018-20075-z (PMC5785970; doi:10.1038/s41598-018-20075-z)
Supplement: Supplementary file 1 — Supplementary Information [file 41598_2018_20075_MOESM1_ESM.pdf]

Supplementary Information

Impact of the huge 2011 Tohoku-oki tsunami on the phenotypes and genotypes of Japanese coastal threespine stickleback populations

Manabu Kume[1,2,3*], Seiichi Mori[1], Jun Kitano[2], Tetsuya Sumi[4] & Shotaro Nishida[1]

[1] Gifu-keizai University, Kitakata 5-50, Ogaki, Gifu 503-8550, Japan, [2] Division of Ecological Genetics, National Institute of Genetics, Yata 1111, Mishima, Shizuoka 411-8540, Japan, [3] Present: Field Science Education and Research Center, Kyoto University, Kitashirakawa-Oiwake, Sakyo, Kyoto, Kyoto 606-8502, Japan, [4] Daido University, Hakusui 40, Minami, Nagoya, Aichi 457-8532, Japan.

*Correspondence and requests for materials should be addressed to M.K.
(m_kume_3ss_es@yahoo.co.jp)

Location*	Year	EC (mS/m)	WT (ºC)	pH
G1	2012	8.3 (6.5-9.1)	11.9 (11.2-12.8)	7.27 (7.19-7.34)
	2013	9.0 (6.5-15.4)	11.8 (10.0-14.3)	7.54 (6.03-8.78)
	2014	7.6 (6.7-8.8)	11.7 (10.4-12.9)	6.89 (6.53-7.26)
G2	2010	9.9 (9.3-11.2)	10.3	6.80
	2011	32.6 (13.5-71.9)	13.9 (10.3-17.4)	6.65 (6.60-6.70)
	2012	9.2 (7.4-11.7)	13.3 (12.1-14.7)	7.09 (6.95-7.27)
	2013	9.8 (6.9-18.7)	12.6 (9.1-18.9)	8.16 (6.64-10.09)
	2014	7.6 (6.9-8.7)	10.9 (9.7-14.0)	7.54 (6.82-8.27)
N1	2011	52.6 (39.3-65.9)	15.9 (15.0-16.8)	5.83 (5.36-6.30)
	2012	165.2 (72.2-315.0)	14.1 (9.6-17.7)	6.79 (6.76-6.83)
	2013	59.2 (14.5-235.0)	13.8 (6.3-21.1)	7.56 (6.22-8.88)
	2014	135.3 (34.9-219.0)	9.0 (6.7-14.9)	7.04 (6.51-7.57)
N2	2012	146.4 (70.9-258.0)	15.8 (13.1-19.2)	6.72
	2013	45.2 (15.1-114.7)	12.3 (6.8-15.2)	7.18 (6.14-8.41)
	2014	52.6 (47.1-56.0)	9.9 (7.6-15.4)	7.45 (6.54-8.35)
N3	2011	65.3 (32.6-145.6)	15.8 (15.1-16.4)	6.38 (6.30-6.45)
	2012	15.1 (7.7-30.7)	14.5 (9.5-18.9)	6.83 (6.58-7.07)
	2013	11.9 (7.1-17.6)	12.9 (7.0-16.5)	7.23 (6.32-8.31)
	2014	8.2 (6.4-10.1)	10.0 (8.5-12.6)	7.23 (6.41-8.04)
N4	2012	9.7 (8.4-11.0)	13.1 (12.0-14.4)	6.79 (6.64-6.93)
	2013	10.7 (7.1-24.1)	12.5 (7.5-14.8)	7.29 (6.32-8.36)
	2014	8.6 (7.2-10.4)	9.6 (7.8-12.5)	7.27 (6.31-8.23)
P1	2012	53.8 (6.6-148.1)	13.5 (11.8-17.1)	7.96 (6.85-9.08)
	2013	63.4 (6.9-299.0)	14.6 (9.0-22.9)	8.18 (7.05-8.88)
	2014	42.4 (6.9-99.6))	8.9 (5.4-15.6)	8.11 (7.21-9.29)
P2	2012	98.9 (79.3-122.0)	15.7 (12.9-18.8)	8.70 (8.68-8.71)
	2013	59.2 (9.6-220.0)	12.7 (8.7-17.1)	9.11 (8.36-10.72)
	2014	28.4 (12.5-56.5)	9.3 (7.1-15.8)	8.69 (7.25-10.13)

Table S1. Summary of environmental factors (means) in each sampling site. Numbers in parentheses indicate the ranges of the environmental parameters. *Abbreviations are shown in the Materials and Methods of the main text, and the locations are shown in Figure 1b**.**

	n	SL	HL	BD	CD	ED	2DSL	LPSL	GRN
Gensui									
1998	20	51.59	16.05	12.22	1.80	4.74	5.44	8.15	20.75
		(4.60)	(1.74)	(1.24)	(0.19)	(0.19)	(0.59)	(0.87)	(1.62)
2011	20	50.82	15.78	12.05	1.88	4.36	5.40	8.30	21.00
		(7.23)	(2.15)	(1.55)	(0.27)	(0.43)	(0.53)	(0.91)	(1.52)
2012	28	46.83	14.22	11.97	1.89	4.33	4.39	6.73	19.04
		(6.05)	(1.88)	(1.68)	(0.34)	(0.38)	(0.64)	(0.93)	(1.20)
2013	20	56.95	17.25	14.99	2.10	4.87	5.61	8.80	19.10
		(4.67)	(1.81)	(1.48)	(0.18)	(0.48)	(0.59)	(0.91)	(1.25)
Namaisawa									
2010	41	56.73	17.70	14.07	2.18	4.91	5.32	8.37	20.39
		(9.54)	(2.53)	(2.62)	(0.41)	(0.49)	(1.25)	(1.49)	(1.84)
2011	20	46.84	14.46	12.22	1.82	4.22	4.91	7.92	20.50
		(2.42)	(0.95)	(0.94)	(0.17)	(0.25)	(0.40)	(0.63)	(1.64)
2012	26	52.81	16.22	14.78	2.11	4.57	4.80	7.60	19.00
		(3.52)	(1.40)	(1.21)	(0.22)	(0.42)	(0.85)	(0.98)	(1.33)
2013	20	52.34	15.84	13.82	1.99	4.39	4.84	8.14	19.80
		(7.22)	(2.23)	(2.05)	(0.30)	(0.51)	(0.85)	(1.12)	(1.01)
New-pool									
2012	18	37.91	10.92	9.51	1.50	3.48	4.25	6.28	21.28
		(2.07)	(0.59)	(0.56)	(0.15)	(0.19)	(0.46)	(0.56)	(1.71)
2013	20	51.61	15.92	13.39	2.03	4.66	4.72	7.65	20.75
		(2.69)	(1.20)	(1.21)	(0.15)	(0.32)	(0.50)	(0.71)	(1.29)
Akkeshi									
JS*	26	64.40	18.91	13.86	2.06	5.17	7.71	9.97	23.38
		(3.79)	(1.44)	(0.93)	(0.19)	(0.32)	(0.65)	(0.83)	(1.63)

Table S2. Summary of morphological data: mean value and standard deviation. These data were used for PC analysis. Specimens in 1998 and 2010 were collected before the tsunami and those in 2011–2013 were collected after the tsunami. *n*: sample size, SL: standard length, HL: head length, BD: body depth, CD: caudal depth, ED: eye diameter, 2DSL: second dorsal spine length, LPSL: left pelvic spine length, GRN: gill raker number.*JS denotes anadromous populations of *Gasterosteus nipponicus* caught in 2003.

	df	Sum of squares	Mean squares	*F*-value	*P*-value
PC1					
Population	1	24.44	24.44	24.07	< 0.0001
Year	3	115.64	38.55	37.96	< 0.0001
Site × Year	3	15.02	5.01	4.93	0.0026
Residuals	187	189.89	1.02		
PC2					
Population	1	0.15	0.146	0.092	0.7619
Year	3	34.28	11.427	7.195	0.0001
Site × Year	3	2.68	0.895	0.563	0.6398
Residuals	187	296.99	1.588		
PC3					
Population	1	4.01	4.01	4.04	0.0459
Year	3	51.83	17.275	17.407	< 0.0001
Site × Year	3	4.99	1.664	1.676	0.1736
Residuals	187	185.59	0.992		

Table S3. Summary of a linear model analysis of the Gensui and Namaisawa populations.

Marker	Gensui				Namaisawa				New pool	
	2010	2011	2012	2013	2010	2011	2012	2013	2012	2013
Stn90	4.65	3.16	3.89	4.05	4.72	4.55	3.59	3.00	3.53	3.00
Stn64	4.99	5.97	5.88	6.52	6.44	6.74	4.99	5.93	6.41	6.94
Stn159	5.66	4.37	4.19	4.32	5.86	5.25	3.68	4.94	4.32	4.94
Stn46	3.44	2.84	2.98	3.29	5.76	5.46	2.00	3.00	3.66	4.00
Stn120	3.00	2.91	2.47	3.36	5.27	5.60	3.90	3.00	4.06	3.00
Stn384	4.49	5.24	5.04	5.72	6.87	7.03	4.98	4.94	4.99	5.00
Stn332	5.17	5.20	4.86	4.25	4.71	5.47	3.99	4.94	3.93	2.00
Stn278	1.75	1.70	1.00	1.61	1.00	1.31	1.00	2.94	2.78	3.00
Stn76	3.25	2.91	3.46	3.37	2.65	2.98	2.00	2.00	2.00	2.00
Stn170	2.75	3.47	3.69	2.77	5.76	5.73	2.00	2.00	3.55	4.87
Stn175	4.96	5.94	5.32	5.24	8.02	8.16	4.97	5.94	4.69	6.87
Stn301	4.25	3.47	4.19	4.23	6.99	7.00	3.58	5.81	5.00	7.81
Stn389	9.03	7.37	9.44	6.99	10.2	10.8	7.55	9.66	9.01	11.8
Stn25	4.07	5.83	4.19	5.40	6.56	6.10	4.43	4.00	4.68	7.75
Stn35	4.49	5.25	4.46	4.38	6.79	6.98	4.67	5.00	4.55	5.94
Ave.	4.40	4.37	4.34	4.37	5.84	5.94	3.82	4.47	4.44	5.26
SE	0.43	0.41	0.48	0.37	0.56	0.56	0.42	0.51	0.42	0.69

Table S4. Allelic richness of each microsatellite locus. Specimens in 1998 were collected before the tsunami and those in 2011–2013 were collected after the tsunami.

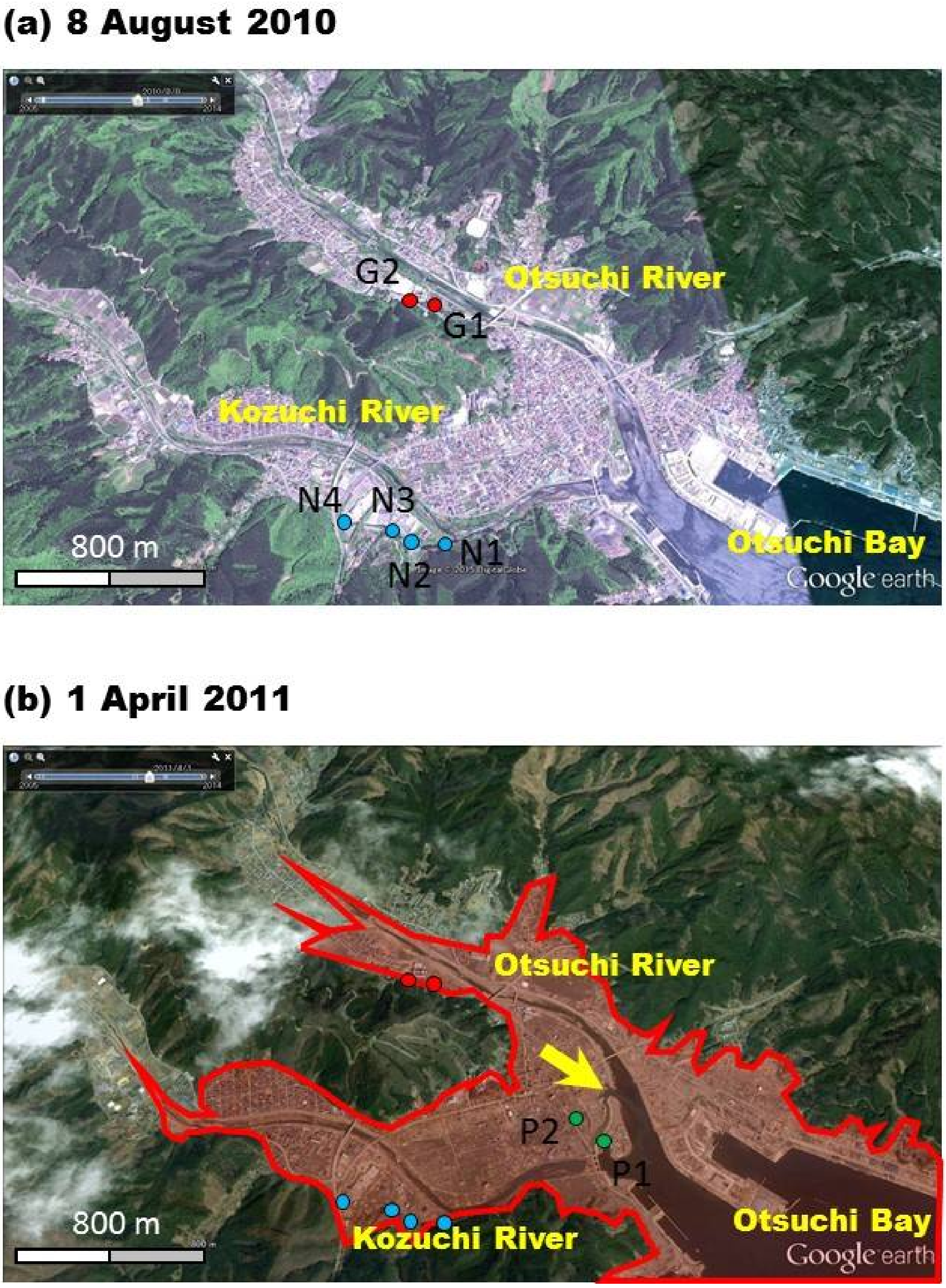

Figure S1. Landscape changes in an urban area of Otsuchi Town before and after the 2011 tsunami triggered by the Tohoku Earthquake off the Pacific coast (11 March 2011). Maps on (**a**) August 8, 2010 (before) and (**b**) April 1, 2011 (three weeks later). The red shaded area indicates the damaged area, where we surveyed on April 5, 6, 29, and 30 and May 1 and 2, 2011. The arrow denotes a broken point of a river levee, from where waters flooded into the urban area. Aerial photographs used in this study were taken from Google Earth (ver. 7.1.8.3036, Map data: Google, Digital Globe).

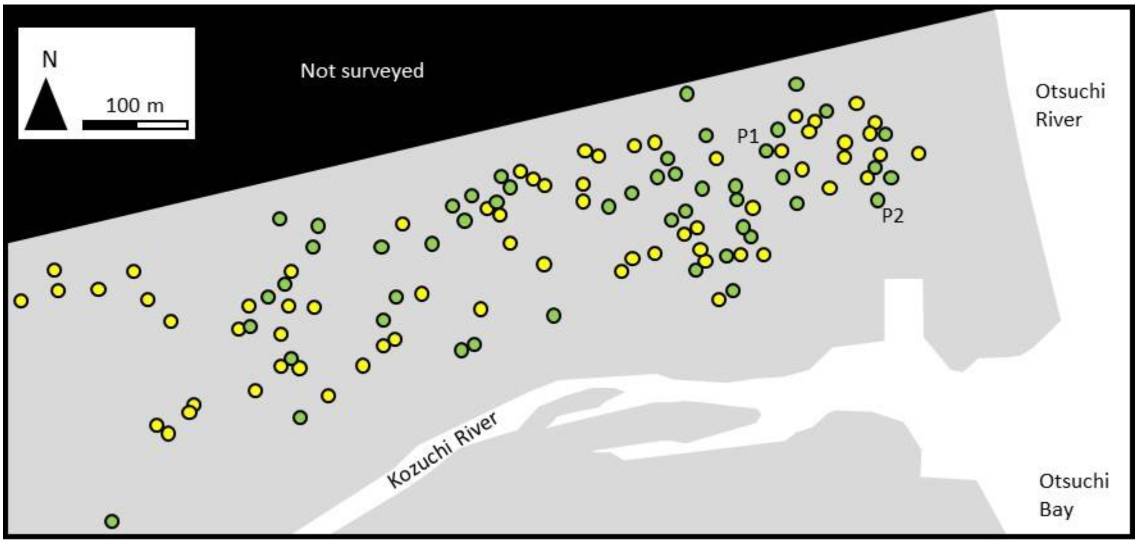

Figure S2. Distribution of flowing wells (circles) in the southern urban area of Otsuchi Town. Green and yellow circles indicate the presence or absence of threespine sticklebacks, respectively. We found sticklebacks in pools and channels around 48 out of 114 flowing wells on August 18, 2014. We could not survey the northern urban area because an embankment for house building had been already started. Map was taken from Garmin BaseCamp (ver. 4.6.2; http://www.garmin.com/en-US/shop/downloads/basecamp)

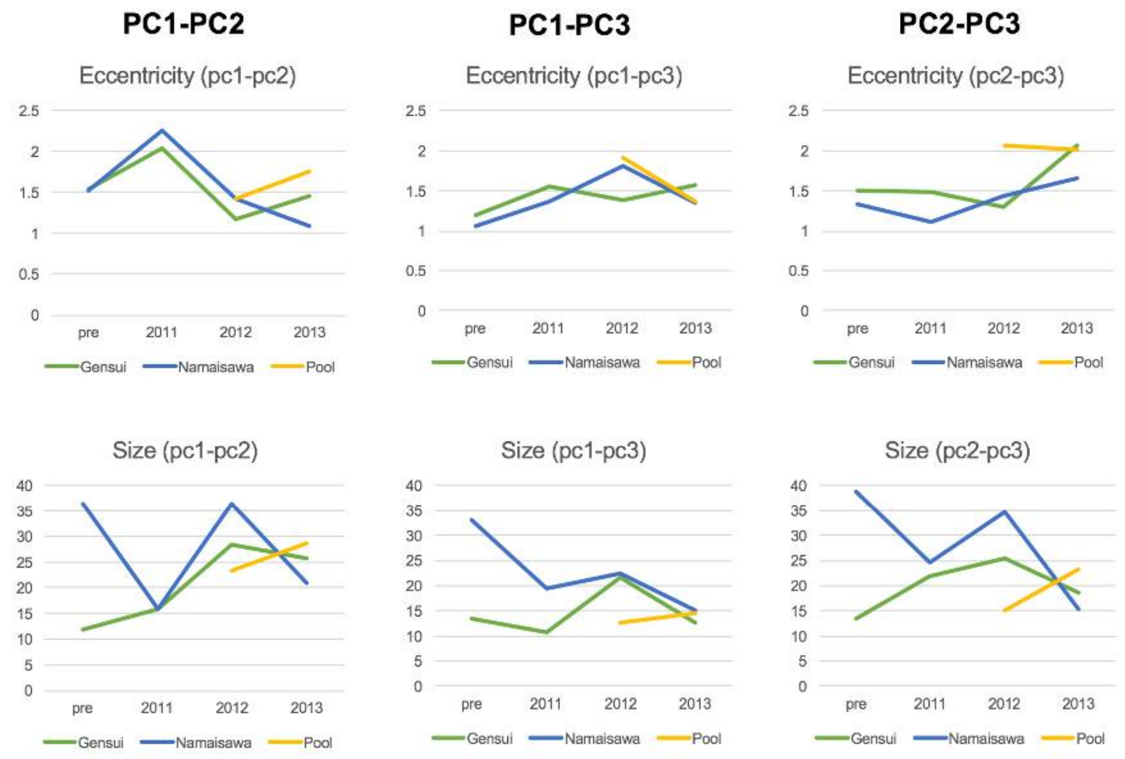

Figure S3. Comparison of the eccentricity and the size of the 95% confidence ellipse of morphological traits.

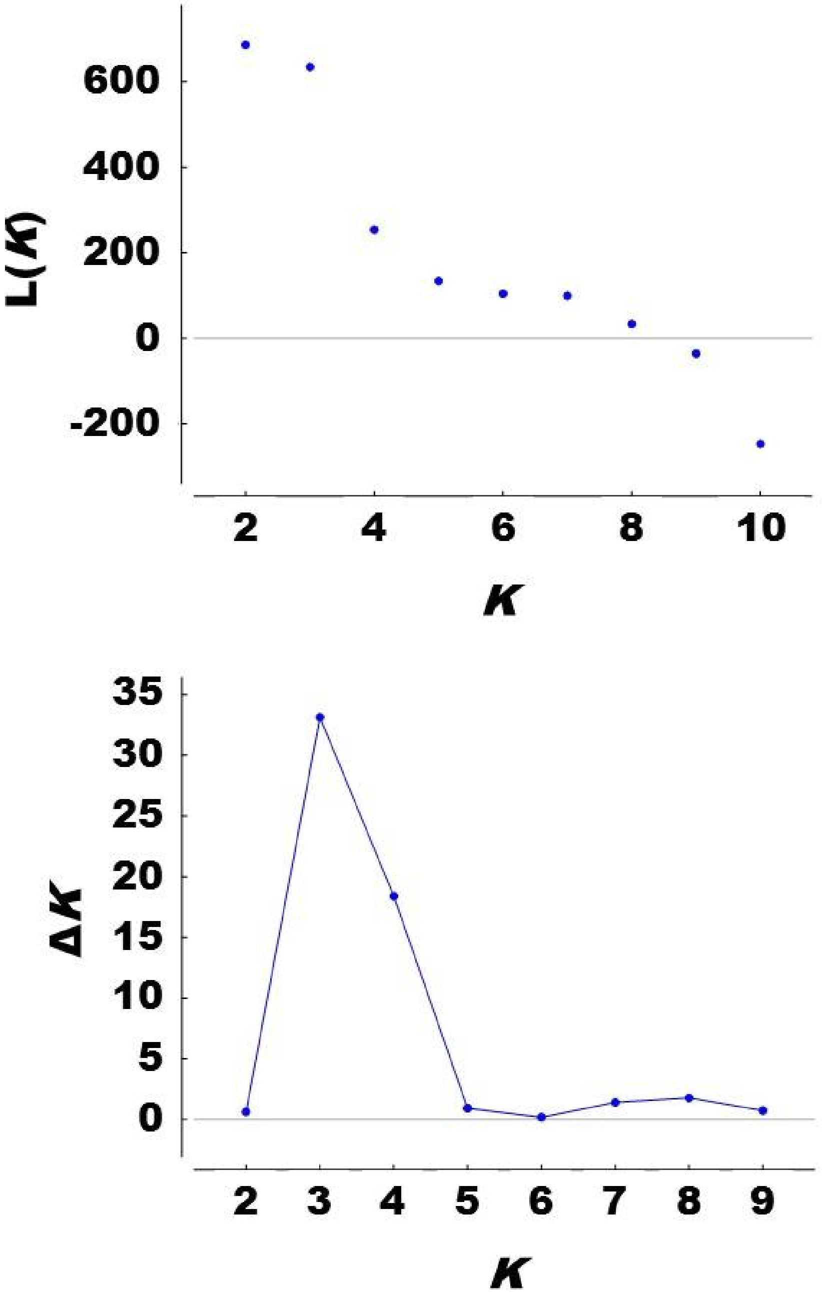

Figure S4. Number of genetic clusters of threespine sticklebacks in Otsuchi Town. The estimation of the genetic cluster number (*K*) by the STRUCTURE software was performed on sticklebacks collected from three sites (G2, N3, and P1) from 2010 to 2013. Three simulations were conducted for $K = 1$ through $K = 9$. The mean ± SD of the log probability of data L(*K*) is shown in the upper panel, and the ad hoc statistic ΔK is shown in the lower panel. The plateau of L(*K*) and the peak of ΔK occur at $K = 3$, suggesting that three is the most probable number of genetic clusters.
